# Tracking Sweet Potato Leaf Curl Virus through Field Production: Implications for Sustainable Sweetpotato Production and Breeding Practices

**DOI:** 10.3390/plants13091267

**Published:** 2024-05-02

**Authors:** Sharon A. Andreason, Petrina McKenzie-Reynolds, Kaitlyn M. Whitley, John Coffey, Alvin M. Simmons, Phillip A. Wadl

**Affiliations:** United States Department of Agriculture, Agricultural Research Service, U.S. Vegetable Laboratory, 2700 Savannah Hwy., Charleston, SC 29414, USA; petrina.reynolds@usda.gov (P.M.-R.); kaitlyn.whitley@usda.gov (K.M.W.); john.coffey@usda.gov (J.C.); alvin.simmons@usda.gov (A.M.S.)

**Keywords:** vegetative propagation, Convolvulaceae, whitefly, begomovirus, digital PCR, *Ipomoea batatas*

## Abstract

Sweet potato leaf curl virus (SPLCV) is a whitefly-transmitted begomovirus infecting sweetpotato and other morning glory (Convolvulaceae) species worldwide. The virus is widespread at the USDA, ARS, U.S. Vegetable Laboratory (USVL), and testing of germplasm maintained in the breeding program indicates nearly 100% infection in storage roots of materials propagated for at least four years. Prior to the public release of new germplasm, viruses must be eliminated via laborious and time-consuming meristem-tip culture. The identification of virus-free seedlings early in the selection process can offer an alternative to meristem-tip culture. In this study, we investigated the transmission of SPLCV over two years of consecutive field plantings (early and late) of sweetpotato. While SPLCV is endemic at the USVL, virus transmission pressure over the typical cultivation season is unknown, and avoidance of virus transmission paired with the selection and maintenance of clean material may be a viable alternative to virus elimination. In 2022, the storage roots of 39 first-year seedling (FYS) selections were tested for SPLCV after early-season cultivation, revealing a single selection (2.6%) with a positive test. Similar testing was conducted in 2023 with no SPLCV-positive FYS selections detected. To further assess SPLCV acquisition in the field, replicated late-season plantings of each selected FYS (n = 37) were monitored from planting to harvest. Testing was conducted at 60 and 120 days after planting (DAP). Approximately 35% of the bulk samples were infected at 60 DAP, and infection increased to 52.3% by 120 DAP. Testing of individuals within selected positive bulked samples did not support 100% infection at harvest. Altogether, these results demonstrate that SPLCV transmission during early planting is sufficiently low to facilitate the maintenance of virus-free selections, offering an alternative to virus cleaning and a cultivation strategy that may be leveraged for production.

## 1. Introduction

Sweetpotato (*Ipomoea batatas*) plays an important role in maintaining food security worldwide. It serves as a staple crop providing a source of carbohydrates, energy, and phytonutrients for human consumption and animal feed [1]. More than 8 million hectares of land are used to produce 89 million tons of sweetpotato globally. Sweetpotato is a good source of vitamins, minerals, and dietary fiber and can be an affordable food source to combat nutritional deficiencies in developing countries [1,2]. Production of sweetpotato relies on vegetatively propagated planting stock (storage roots and slips) that can accumulate and serve as inoculum for the dissemination of phytopathogenic viruses. Accumulation of viruses can severely decrease yield and quality in vegetatively propagated sweetpotato. Over 30 different viruses are known to infect sweetpotato, with many of these being vector-transmitted by whiteflies or aphids [3].

Sweet potato leaf curl virus (SPLCV; Geminiviridae: Begomovirus) is one of the most economically important pathogens infecting sweetpotato on a global scale. Sweetpotato and other Convolvulaceae-infecting begomoviruses make up a monophyletic group within the begomoviruses called sweepoviruses [4]. The sweepoviruses have monopartite, circular, single-stranded DNA genomes sometimes associated with DNA satellites [5,6,7]. Like other begomoviruses, the sweepoviruses are vector-transmitted exclusively by the sweetpotato whitefly, *Bemisia tabaci* (Hemiptera: Aleyrodidae), in a persistent-circulative manner [8,9]. Management of whiteflies is fundamental to the prevention of SPLCV introduction to virus-free sweetpotato propagative materials. However, whitefly control is notoriously challenging in conducive field environments given *B. tabaci*’s high fecundity, short life cycle, and resistance to insecticides, as well as the limited effectiveness of biological control agents against *B. tabaci* [10].

SPLCV has been identified in many major sweetpotato growing regions globally and has a wide host range within *Ipomoea* [3,11]. Host range studies revealed that over 80% of tested *Ipomoea* spp. were susceptible hosts of both SPLCV and *B. tabaci* [11,12]. These *Ipomoea* spp. can serve as potential reservoirs for the reinfection of virus-indexed sweetpotato planting stock. Symptoms of SPLCV can include upward curling of leaf margins, stunting, yellowing, and yield loss, but symptoms are not always present, can fade as the plant matures, and are host species- or cultivar-dependent [3,13,14]. Yield losses due to SPLCV can be significant, ranging from 10 to 80%, depending on the cultivar [13]. Rouging of nearby reservoirs and host plant resistance are critical to preventing the spread of SPLCV.

The production of sweetpotato, whether for large-scale commercial production or in low-input, subsistence farming practices, utilizes vegetatively propagated planting stock (storage roots and slips) [3]. As SPLCV moves systemically through the plant and accumulates in the storage roots, vegetative planting stock may be infected with the virus, effectively limiting sweetpotato production. Planting of virus-free slips is then critical to maintaining high-yield production, but clean propagative materials can be challenging, if not impossible, to sustain through successive cycles of production in areas with high vector and virus pressure. However, seeds are a source of virus-free propagation due to a lack of definitive evidence for seed transmission of sweetpotato viruses [3,15,16]. Seedlings are reliably SPLCV-free due to a lack of seed transmission of the virus, as demonstrated by a previous study [16].

In the Sweetpotato Breeding Program housed at the USDA, ARS, U.S. Vegetable Laboratory (USVL) in Charleston, SC, USA, sweetpotatoes are generated from first-year seedlings (FYS) and then maintained vegetatively for a minimum of 3–4 years before being selected as a superior genotype and advanced to public release. This duration of time in the field leads to most if not all, selections having acquired viruses, particularly SPLCV. Before the public release of a well-performing cultivar, any viruses present must be eliminated via meristem-tip culture [3]. As many as three years can be dedicated to generating virus-indexed plant material needed for the production of planting stock for large-scale distribution to growers. The identification of virus-free plants within the first year of selection via a combination of molecular and hyperspectral imaging methods could be used to remove this bottleneck from the cultivar development pipeline.

SPLCV is the most prevalent sweetpotato virus at the USVL and has been demonstrated to rapidly reinfect virus-indexed planting stock and significantly decrease yield in sweetpotato cultivars [13]. FYS are expected to be virus-free prior to field planting, as SPLCV is not seed-transmitted [16]. The planting of virus-indexed materials could permit the harvest of virus-free selections before the end of the growing season if SPLCV transmission is sufficiently low during early planting when the first round of selections is made. Given our previous observations of low vector pressure throughout early-season planting compared to late-season planting, we hypothesize that whitefly transmission of SPLCV in early plantings will be low enough to allow the harvest and maintenance of virus-free selections. The goal of this study was to determine the level of SPLCV infection that occurs naturally in sweetpotato FYS during the growing season, specifically at early selection through late-season planting. Two years of breeding cycles were evaluated. Plants were tested for SPLCV infection after an early-season planting in year 1 and throughout a late-season planting in year 2. Our results demonstrate that early-season FYS planting can largely avoid the threat of SPLCV and likely other vector-transmitted viruses due to a low abundance of vectors compared to late-season. Viral acquisition within the seedlings can be low enough to allow alternative methods to be developed for eliminating the laborious and time-consuming process of virus elimination via meristem-tip culture. Strategically leveraging this agroecological phenomenon can impact sweetpotato breeding, the discovery and development of host plant resistance, and production in both conventional and low-input cropping systems.

## 2. Results

**Figure 1:**
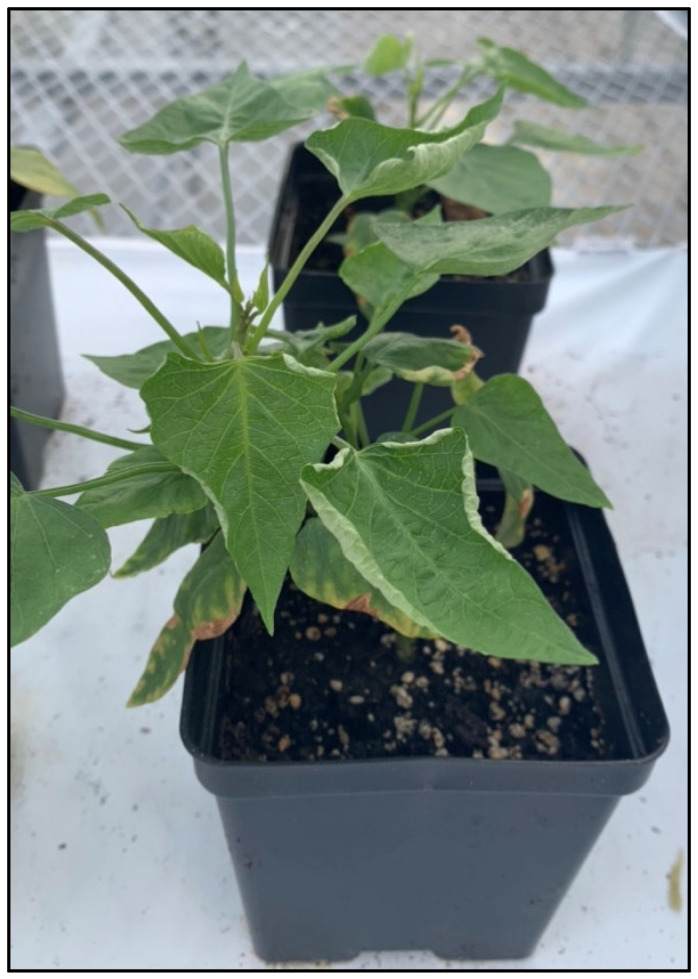
Sweet potato leaf curl virus (SPLCV) infection in sweetpotato (*Ipomoea batatas*) planted in a 4-inch square pot. Characteristic upward curling of leaf margins is demonstrated. This symptom is typically the only aboveground indication of SPLCV infection and is commonly short-lived in younger plants and fades as the plant matures.

SPLCV (Figure 1) infection was tracked in sweetpotato FYS through two cycles of a breeding program. In the first year (2022), 39 FYS were selected for having an erect or semi-erect plant habit and satisfactory storage root traits after 8 weeks of early-season cultivation. Of the 39 FYS selection root samples tested, only one selection was positive for SPLCV (Table 1). Whitefly vector pressure was low throughout early-season cultivation in year 1.

In the second year (2023), sweetpotato was monitored for SPLCV infection at the transition from early-season to late-season planting (81 days after planting [DAP] early-season/0 DAP late-season), 60 DAP of the late-season planting, and at late-season harvest (120 DAP). At 81 DAP of early-season cultivation (April–July), 37 FYS were selected for desirable plant habit and storage root traits. Of the 37 selections, none of the FYS leaf tissue samples were positive for SPLCV (Table 1). Whitefly pressure was again low during early-season cultivation. At 60 DAP in late-season cultivation (mid-September), 35.2% of bulked leaf tests were positive for SPLCV (Table 1). Bulked tests included up to 10 individual plant leaf tissue samples; one (or more) SPLCV-positive plants out of 10 bulked plants could produce a positive test. At harvest (November), the FYS genotypes that had not acquired an SPLCV infection by mid-season were tested along with selected positive control FYS that were infected by mid-season. An additional 19 bulked replicated genotypes were positive at harvest, resulting in 52.3% of FYS samples infected with SPLCV by late-season harvest (Table 1).

To estimate the percentage of individual plants that were infected with SPLCV, individual plants of 10 randomly selected FYS genotypes were tested. Two to 10 plants per genotype were available for testing. Of 80 FYS plants tested, 25 (31.3%) were positive for SPLCV (Table 2). A subset of these individuals was selected to confirm the acquisition of SPLCV into the storage roots. Tests confirmed that SPLCV was also detected in the storage roots of plants that tested positive for the virus in the leaf tissue.

## 3. Discussion

Sweet potato leaf curl virus is the most economically important whitefly-transmitted virus affecting sweetpotato in the U.S. With the global distribution of its invasive vector, *B. tabaci*, this virus is a threat to sweetpotato production worldwide. Sweetpotato production practices vary within the U.S. and globally, making breeding for broad-spectrum host resistance critical, particularly for low-input and subsistence farming practices. Currently, breeding and selection of superior sweetpotato genotypes, followed by the elimination of viruses prior to public release, is a 5–10-year process that could be reduced via strategic breeding practices.

Vector-transmitted viruses constitute one group of selective pressures present at the USVL, enhancing the development of durable, broad-spectrum-resistant sweetpotato cultivars. Whitefly-transmitted SPLCV is abundant in reservoir *Ipomoea* species in the area and often infects all selections by year 4 of maintaining breeding selections. Aphid-transmitted potyviruses, including sweet potato virus G, sweet potato virus C, sweet potato feathery mottle virus, and sweet potato virus 2, are also present. This study focused on SPLCV due to its abundance in nearby reservoirs and its presence in all selections (100%) advanced through four generations of propagation within the USVL sweetpotato breeding program. The aforementioned potyviruses have been detected in selections advancing to virus elimination (after ~4–5 years of cultivation and selection) but in only up to 25% of submitted clones.

Sweetpotato breeding at the USVL has been ongoing for over 50 years and has a history of releasing insect and root-knot nematode-resistant germplasm that has been utilized in commercial cultivation and as parental germplasm in public breeding programs around the globe. The goal of our study was not to breed resistance to SPLCV; rather, it was to determine if early planting to harvest of advanced selections is a viable option for the maintenance of virus-free selections. However, in terms of sweetpotato breeding, due to the rapid infection and reinfection of germplasm maintained in the USVL breeding program, we may unintentionally select for tolerance or resistance to SPLCV. SPLCV can drastically lower yield in sweetpotato [13], and because of the multi-year evaluation process that is needed before germplasm is released, susceptible genotypes may be discarded due to low yield. The major goals of the USVL sweetpotato breeding program are to develop insect and nematode resistance, and our study was designed to develop a method to allow sweetpotato breeders to utilize seasonal variation in whitefly presence to minimize SPLCV acquisition and reduce labor and time in releasing virus-free sweetpotato cultivars through the elimination of the need for virus cleaning via meristem culture. However, utilizing a planting strategy to avoid the seasonality of viral vectors would allow sweetpotato breeders to discern antagonistic pleiotropic responses to SPLCV by providing a virus-free clonal propagule that can be compared with a virus-infected propagule of the same genotype in side-by-side field studies to elucidate the impact of virus infection on yield.

In two years of the program, early FYS plantings were monitored for vector presence and then tested for SPLCV at selection prior to late-season slip planting. At this phase in the breeding year, selected storage roots may be stored for planting stock. In year 1, only one plant out of 39 selections (2.6%) was positive for SPLCV, and in year 2, no selections out of 37 had SPLCV. In both years, whiteflies were in low abundance throughout the early planting, spanning May into early July. The late-season planting (monitored/tested in year 2) resulted in a rise in vector numbers in July, with the highest abundance in August and September. Slips of selected FYS that were planted for late-season selections in 2023 were free of SPLCV. By 60 DAP, SPLCV had moved into FYS, with detection in 35.2% of bulked leaf samples. By harvest (120 DAP), SPLCV was detected in 52.3% of bulked samples. Taking a closer look at rates of individual plant infections within subplots (up to 10 plants per subplot), testing a random selection of FYS genotypes estimated that 31.3% of FYS plants were infected with SPLCV by harvest. This rate aligns with observations that most, if not all, selections will be infected with SPLCV by year 4 of breeding. Therefore, the early planting of sweetpotato in regions where whitefly populations will be low in spring and early summer can provide effective SPLCV management, even where virus pressure is present throughout the growing season.

In the same vein as our suggestion of strategic planting to evade vector pressure and virus transmission, an observation of a short timeframe for aphid-borne potyvirus transmission has been made. A preliminary study of potyvirus transmission to sweetpotatoes in Louisiana showed that, despite aphid abundance throughout the production season, viruses were transmitted predominantly within a one- to two-month period [3,17]. Although this study indicated potyvirus transmission shortly after slip transplant into the field (whereas FYS remained largely SPLCV-free during early planting in the present study), an argument can be made that altering the planting date to miss this short window of transmission pressure may provide effective management of aphid-transmitted viruses, in addition to whitefly-transmitted virus management.

Avoidance is the foundation of pest and disease management in an integrated pest management strategy and has demonstrated effectiveness for control of *B. tabaci* and whitefly-transmitted viruses [18,19]. Early planting and manipulation of the production season are strategies adopted to avoid heavy late summer whitefly populations for cotton production in the U.S. Southwest and Mexico [19,20,21,22,23]. As we have demonstrated in this study, the incidence of viruses transmitted by *B. tabaci* can build as whitefly populations increase into the late summer. An increase in whitefly abundance in cotton and melon from July through September in southern California was correlated with an increase in incidence of viruliferous whiteflies and lettuce infectious yellows virus, a semi-persistently transmitted crinivirus (Closteroviridae), in melon and lettuce in October and November [24]. Similarly, in a recent survey of whiteflies collected from squash in the southeastern U.S., the incidence of whiteflies carrying the begomoviruses cucurbit leaf crumple virus or sida golden mosaic virus increased from no virus detection in whiteflies collected in early August to a majority of whiteflies carrying one of the two viruses by late August. Successful avoidance of virus transmission by insect vectors in early sweetpotato planting, as demonstrated herein, provides a strategy that may be utilized in commercial production in addition to breeding. Certified virus-free slips can be used to produce foundation rootstock [25], and virus-free slips may be planted in disease-free soil early to avoid pests and virus pressure for the production of clean seed material. Of course, the success of this approach can vary with fluctuations in whitefly populations year to year or seasonally within different growing regions and climates and following different environmental conditions. Climate change may also affect the effectiveness of this strategy over time.

In the present study, we show that storage roots harvested from an early planting of virus-free FYS are unlikely to be infected with vector-transmitted viruses, specifically SPLCV. Early planting of sweetpotato in temperate and many subtropical areas can evade pest and virus pressure, allowing the generation of virus-free planting stock. Clean material can be harvested at the selection of well-performing genotypes and maintained separately in an enclosed environment free of vector and virus pressure throughout advanced selection cycles to negate the need for virus elimination. This strategy of virus evasion may be used in sweetpotato breeding programs or in production as a sustainable means of cultivating clean propagation material.

## 4. Materials and Methods

### 4.1. Generation of First-Year Seedlings in the Sweetpotato Breeding Program

**Figure 2:**
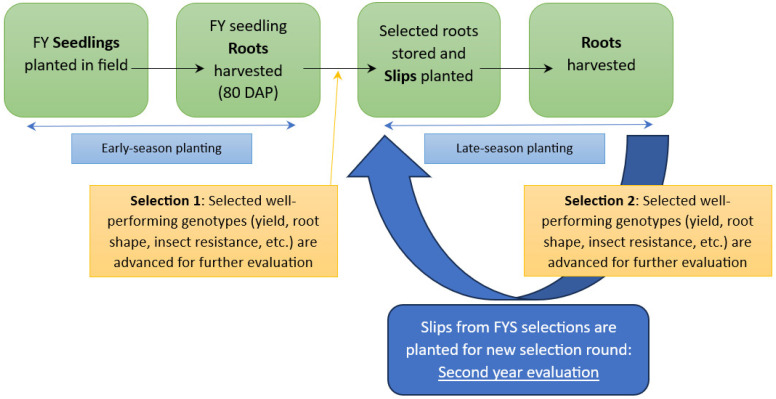
Breeding selection cycle for sweetpotato (*Ipomoea batatas*) used in this study. First-year seedlings (FYS) are planted in the early season and selected ~80 days after planting. At the first selection, slips are prepared from the selected FYS and used for another round of selection in a replicated field test. The storage roots from the early- and late-season are evaluated, and undesirable FYS are eliminated from the breeding program, whereas the storage roots from the FYS advanced for additional evaluation are saved for propagation and evaluation the next season.

The FYS tested in this study were derived from selected sweetpotato germplasm used in open-pollinated crosses within the USVL Sweetpotato Breeding Program. The USVL program uses recurrent mass selection using an open-pollinated polycross system (15–25 parental clones) that relies on natural populations of various insects for cross-pollination [26]. The parents used in the breeding nurseries change each year based on selections made in the previous breeding cycles. The plant material tested in this study is derived from seeds harvested from maternal clones that were included in breeding nurseries. In 2022, an individual slip (~30 cm long) from 4125 FYS derived from 52 maternal parents was planted into narrow beds (~38 cm wide by 30 cm tall) that were prepared by forming soil into rows ~1 m apart. Fertilizer (1121 kg ha^−1^ of 4N-3.5P-10K, Nutrien; Saskatoon, SK, Canada) was incorporated into the soil before bedding. Formed beds were treated with the herbicides clomazone (Command 3ME; FMC Corporation, Philadelphia, PA, USA) at 0.4 kg ai/ha and napropamide (Devrinol 2EC; United Phosphorus, Inc., King of Prussia, PA, USA) at 1.1 kg ai/ha. Slips were planted at a spacing of 76.2 cm on 4 May 2022. When weekly rainfall was not adequate (<2.54 cm) during the growing season, supplemental overhead irrigation was applied until all plots had received a total of 2.54 cm. Approximately 80 DAP, 39 FYS were selected for a combination of vigorous growth with semi-erect to erect plant habit, yield, root shape, consistent flesh color, and insect resistance. In 2023, an individual slip (~30 cm long) from 6200 FYS derived from 59 maternal parents was planted on 20 April 2023 and subjected to the same conditions as in 2022. Using the same selection criteria as in 2022, 37 FYS were selected (Figure 2).

### 4.2. Field Plots and Selections Monitored for SPLCV Infection

**Figure 3:**
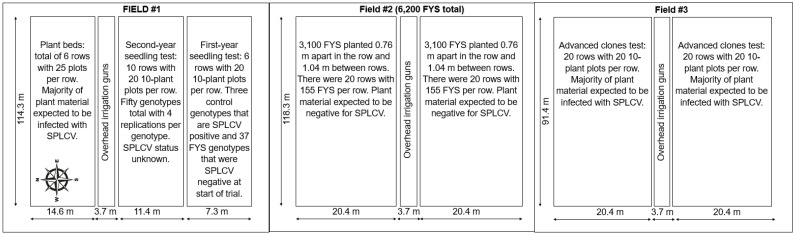
Diagrammatic representation of the layout for sweet potato leaf curl virus (SPLCV) tracking experiments conducted at the USDA, ARS, U.S. Vegetable Laboratory (Charleston, SC, USA) in 2023. Fields #1 and #3 contained plant material (plant beds and advanced clones) infected with SPLCV and provided a natural source of inoculum for tracking infection of first-year seedlings (FYS). The FYS initially grown in field #2 were assumed to be negative for SPLCV based on the results of Andreason et al. [16] and confirmed SPLCV-free by digital PCR testing after selection. At 81 days after planting (DAP), 37 FYS were selected for having an erect to semi-erect plant habit and acceptable storage root traits (shape, skin and flesh color, etc.). Slips were prepared for each FYS selection and used as planting stock in replicated plantings for tracking SPLCV acquisition in field #1. At 0, 60, and 120, DAP bulked leaf tissues were tested for SPLCV. At harvest (125 DAP), randomly selected individual plant leaves and roots were tested for SPLCV.

During the early-season planting in year 1, FYS were planted in the field in May 2022. Immediately north and south of the FYS plot were plant beds containing advanced lines of sweetpotato with mixed infections of SPLCV and other viruses (Figure 3). Eight weeks after planting, roots were harvested, and selections for bunch-type plant habit and satisfactory roots were made. Early-season selection roots (n = 39) were tested for SPLCV (Figure 2).

In year 2, FYS were planted for the early-season planting and primary selection in April 2023 (Figure 2). Eight weeks after planting, primary selections were made, and replicated 10-plant plot plantings (n = 3) of each selected FYS (n = 37) were planted in Field #1 for late-season secondary selections. These selections were screened for SPLCV at three time points: (1) initial planting after early-season selection (0 DAP; 14 July 2023); (2) mid-season (60 DAP; 11 September 2023); and (3) at harvest (120 DAP; 9 November 2023). Additionally, at 125 DAP, randomly selected individual plants (leaf material and roots) were tested for SPLCV. As in year 1, the plot monitored for SPLCV was planted near fields with SPLCV-infected material (Figure 3), and all fields were surrounded by drainage ditches with wild *Ipomoea* spp. growing in the ditches and the margins of the fields. In both years, fields were periodically monitored for the presence of virus vectors, including whiteflies and aphids.

### 4.3. Testing for SPLCV in First-Year Seedling Selections

DNA was extracted from sweetpotato storage roots using the DNeasy Plant Mini Kit (QIAGEN, Hilden, Germany) (Table 1). Approximately 1 cm^2^ of tissue was excised from the storage root and lysed in 2–4 mL AP1 buffer (QIAGEN) in an extraction bag (Bioreba, Reinach, Switzerland) using the Homex 6 homogenizer (Bioreba, Reinach, Switzerland). Extraction was then carried out following the manufacturer’s instructions. DNA was extracted from sweetpotato leaf tissues by one of two methods, either using the DNeasy Plant Mini Kit or the KingFisher Apex System (Thermo Fisher Scientific, Waltham, MA, USA) (Table 1). Using the DNeasy kit, DNA was extracted from leaf tissues (new growth leaves or excision of older leaf tissues down to approximately 1 cm^2^ of the leaf base and petiole) in 2 mL AP1 buffer following the kit instructions. Using the KingFisher Apex, leaf tissues were initially lysed in 2 mL lysis buffer in a Bioreba extraction bag using the Homex 6 homogenizer, and then DNA was extracted using the MagMAX Plant DNA Isolation Kit (Thermo Fisher Scientific, Waltham, MA, USA) following the manufacturer’s instructions. DNA extracts were quantified using a DeNovix DS-11 FX Spectrophotometer/Fluorometer (DeNovix Inc., Wilmington, DE, USA).

SPLCV was detected using Qiacuity digital PCR (QIAGEN). Reactions consisted of 5 µL EvaGreen master mix (QIAGEN), 0.6 µL 10 µM forward and reverse primers (SPLCV.F2/SPLCV.R2; Ling et al. [11]), 1.5 µL sweetpotato DNA at various concentrations (1.0 ng/uL for root tissue extracts; 1–50 ng/uL for leaf tissue extracts), and 7.3 µL nanopure water. Reactions were performed in 96 well nanoplates with 8.5 k partitions per well. Thermocycling consisted of an initial 2 min denaturation at 95 °C, 40 cycles at 95 °C for 15 s and 60 °C for 30 s, and a final extension for 10 min at 35 °C. Imaging was performed at an exposure duration of 200 ms and gain of 6, and the threshold for positive partitions was set at 50 RFU. To interpret digital PCR results and determine positive versus negative tests, the percentages of fluorescing partitions and 1D scatterplots were evaluated (Figure S1). At least 2% of partitions fluorescing in a narrowed range above the threshold on the 1D scatterplot were counted as a positive test. In cases where detection was below the 2% positive partition minimum but not immediately determinant as negative tests, individual tests were reperformed at a range of template DNA concentrations to validate the results. Final positive tests were outlined in blue (Figure S1). Testing of storage roots derived from plants with leaf extract tests resulting in as low as 3% positive partitions confirmed that SPLCV detection was accurate at these low viral titers in leaf tissue extracts.

## 5. Conclusions

Field grown sweetpotato was monitored for the detection of SPLCV over two years of cultivation in a breeding program at the USVL in Charleston, SC, USA. In this study, SPLCV was detected in a single FYS selection in 2022 and in no selections in 2023 at harvest of an early planting. SPLCV was detected at increasing rates over time through late-season planting. The SPLCV vector, *B. tabaci*, was rarely observed in the early planting, whereas the abundance of the vector increased during late planting. Early planting of sweetpotato enabled the avoidance of *B. tabaci* and SPLCV transmission in an area where virus reservoirs are abundant, and vector populations increase in the late summer. Virus-free propagation material may be sustainably produced as part of a breeding program or in production using early planting as a strategy for virus avoidance.

## Figures and Tables

**Table 1:** Evaluation of sweetpotato (*Ipomoea batatas*) first-year seedlings (FYS) generated at the USDA, ARS, U.S. Vegetable Laboratory sweetpotato breeding program (Charleston, SC, USA) for sweet potato leaf curl virus (SPLCV) in 2022 (year 1) and 2023 (year 2). FYS were tested for SPLCV in an early-season planting (May–July) in year 1 and throughout late-season planting (July–November) in year 2.

Test	Year	Season	Days after Planting (DAP)	Tissue	Sampling Method	DNA Extraction Method	No. FYS Samples Tested; Positive Tests	Percent FYS with SPLCV
1	1	Early	56	Root	Bulked	DNeasy	39; 1	2.6%
2	2	Early/Late	81/0	Leaf	Individual	DNeasy	37; 0	0%
3	2	Late	60	Leaf	Bulked	KingFisher	108; 38	35.2%
4	2	Late	120	Leaf	Bulked	KingFisher	76; 19	* Total: 52.3%

* Selected FYS, including all that were negative for SPLCV at 60 DAP plus randomly selected previously positive samples, were tested at 120 DAP. An additional 19 bulked genotypes were positive at 120 DAP, which totaled 52.3% of replicated FYS positive for SPLCV at 60 DAP and/or 120 DAP.

**Table 2:** Evaluation of sweetpotato (*Ipomoea batatas*) first-year seedlings (FYS) generated at the USDA, ARS, U.S. Vegetable Laboratory sweetpotato breeding program (Charleston, SC, USA) for sweet potato leaf curl virus (SPLCV) in 2023 (year 2). FYS leaf and root tissue were tested for SPLCV 125 days after late-season planting (DAP). Ten selected FYS genotypes were tested for SPLCV to estimate the rate of an individual plant infection, and the storage roots of a subset of these selected FYS were tested to confirm the accumulation of SPLCV in the roots.

Test	Year	Season	Days after Planting (DAP)	Tissue	Sampling Method	DNA Extraction Method	No. FYS Samples Tested; Positive Tests	Percent FYS with SPLCV
5	2	Late	125	Leaf	Select Individuals	KingFisher	80; 25	31.3%
6	2	Late	125	Root	Bulked—Select Individuals	DNeasy	14	* As expected

* SPLCV was detected in the storage roots of all virus-positive FYS (leaf tests).

## Data Availability

The data presented in this study are available in Supplementary Materials.

## Supplementary Materials

The following supporting information can be downloaded at https://www.mdpi.com/article/10.3390/plants13091267/s1. Figure S1: Digital PCR results represented as 1D scatterplots with a percentage of positive partitions evaluated for the detection of sweet potato leaf curl virus in six separate tests performed on bulked and individual sweetpotato leaf and storage root samples collected over the course of a sweetpotato breeding program in 2022 and 2023.
