# Supplementary material for: Tracking Sweet Potato Leaf Curl Virus through Field Production: Implications for Sustainable Sweetpotato Production and Breeding Practices
Source: Plants (Basel). 2024 May 2;13(9):1267. doi: 10.3390/plants13091267 (PMC11085579; doi:10.3390/plants13091267)

TRACKING SPLCV – Supplementary Data – QIACUITY SCATTERPLOTS

Figure S1

TEST 1: Early-Season Roots (56 DAP)

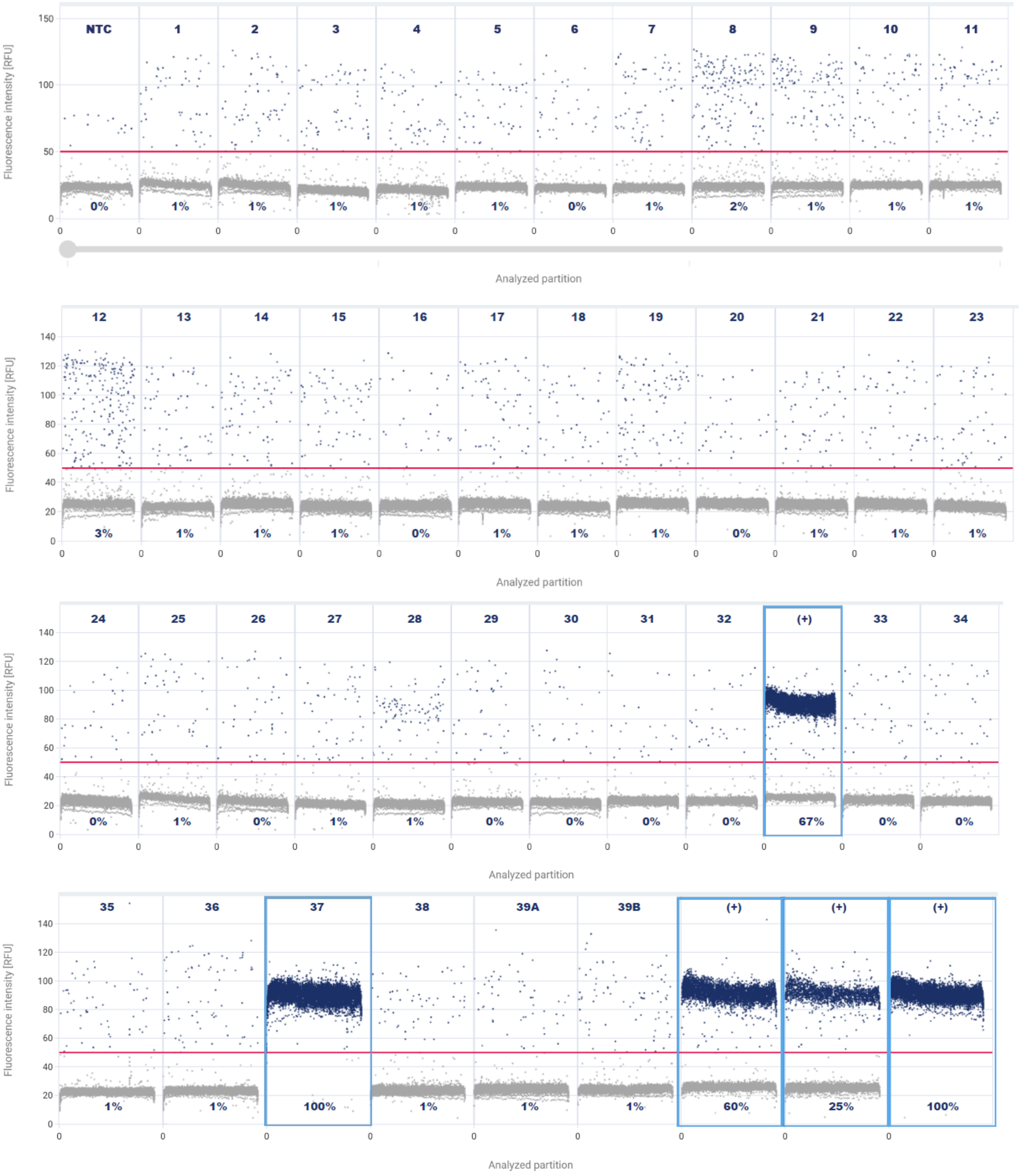

TEST 2: Early/Late-Season Leaves (81/0 DAP)

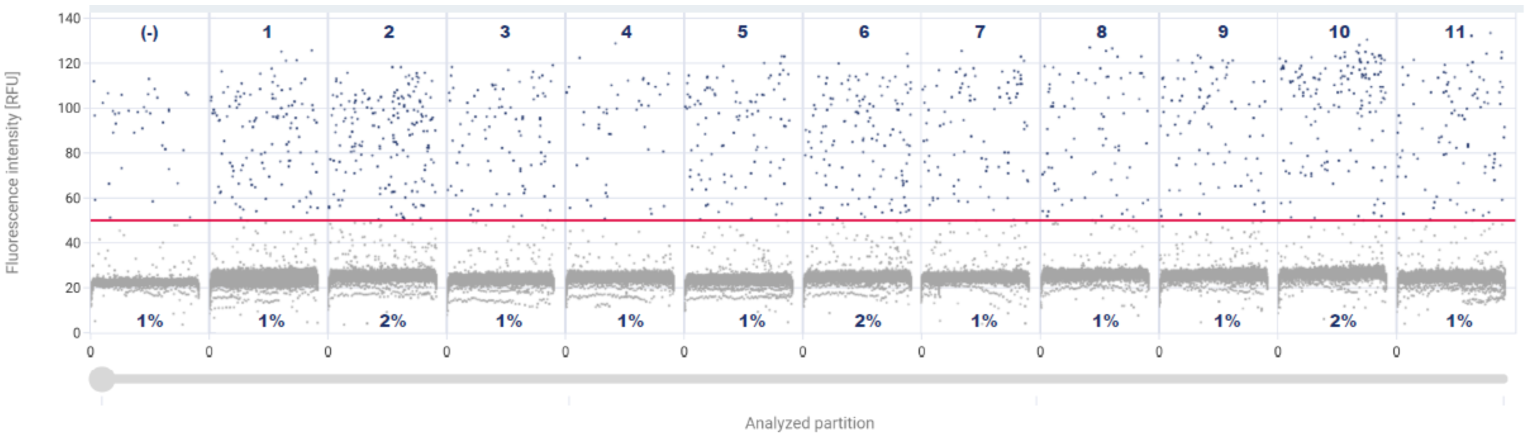

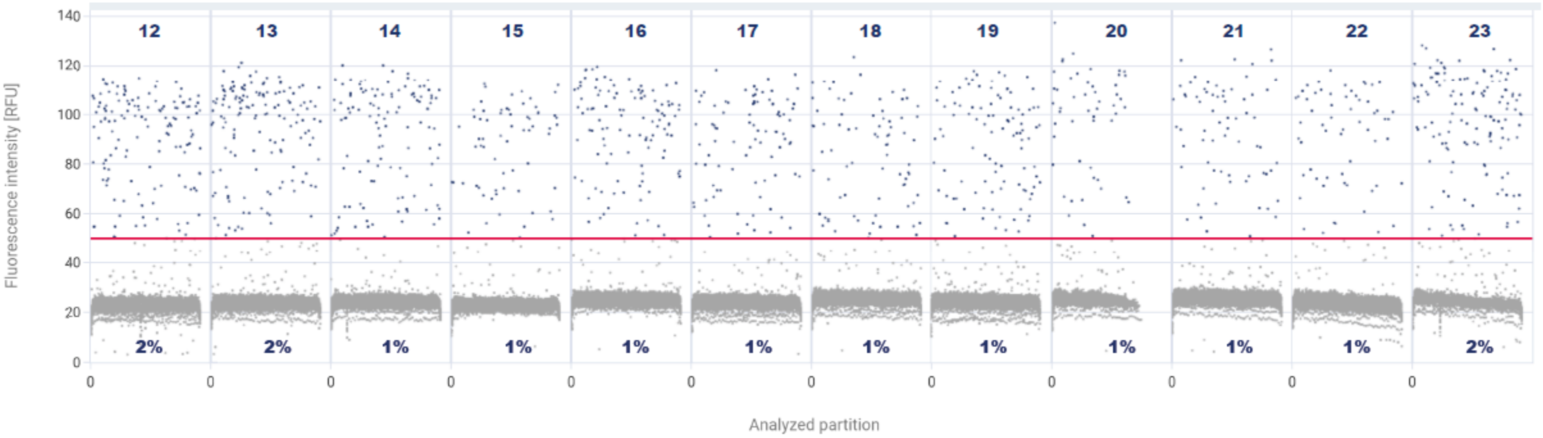

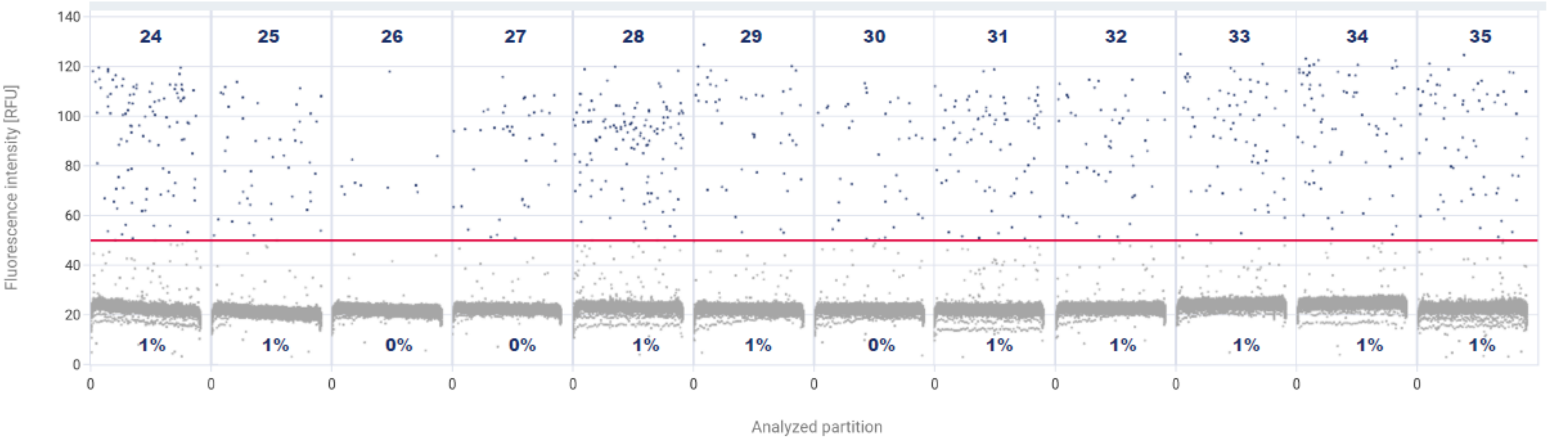

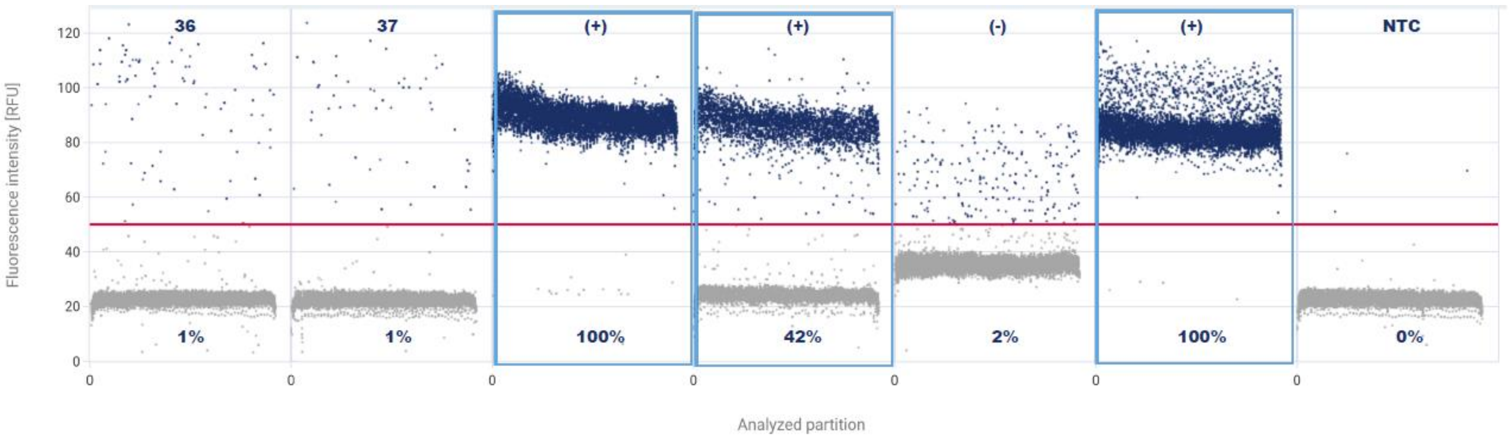

TEST 3: Late-Season Leaves (60 DAP)

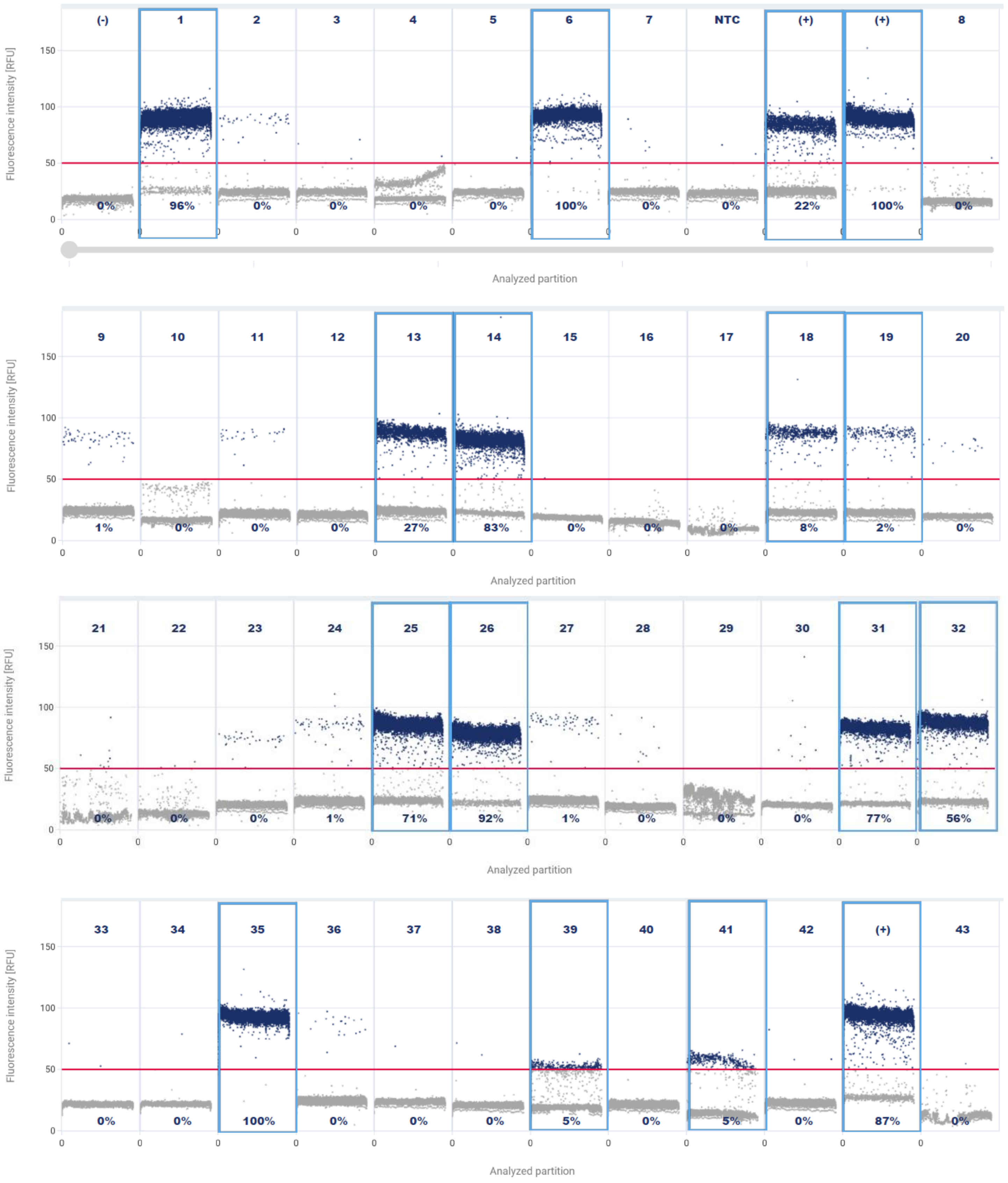

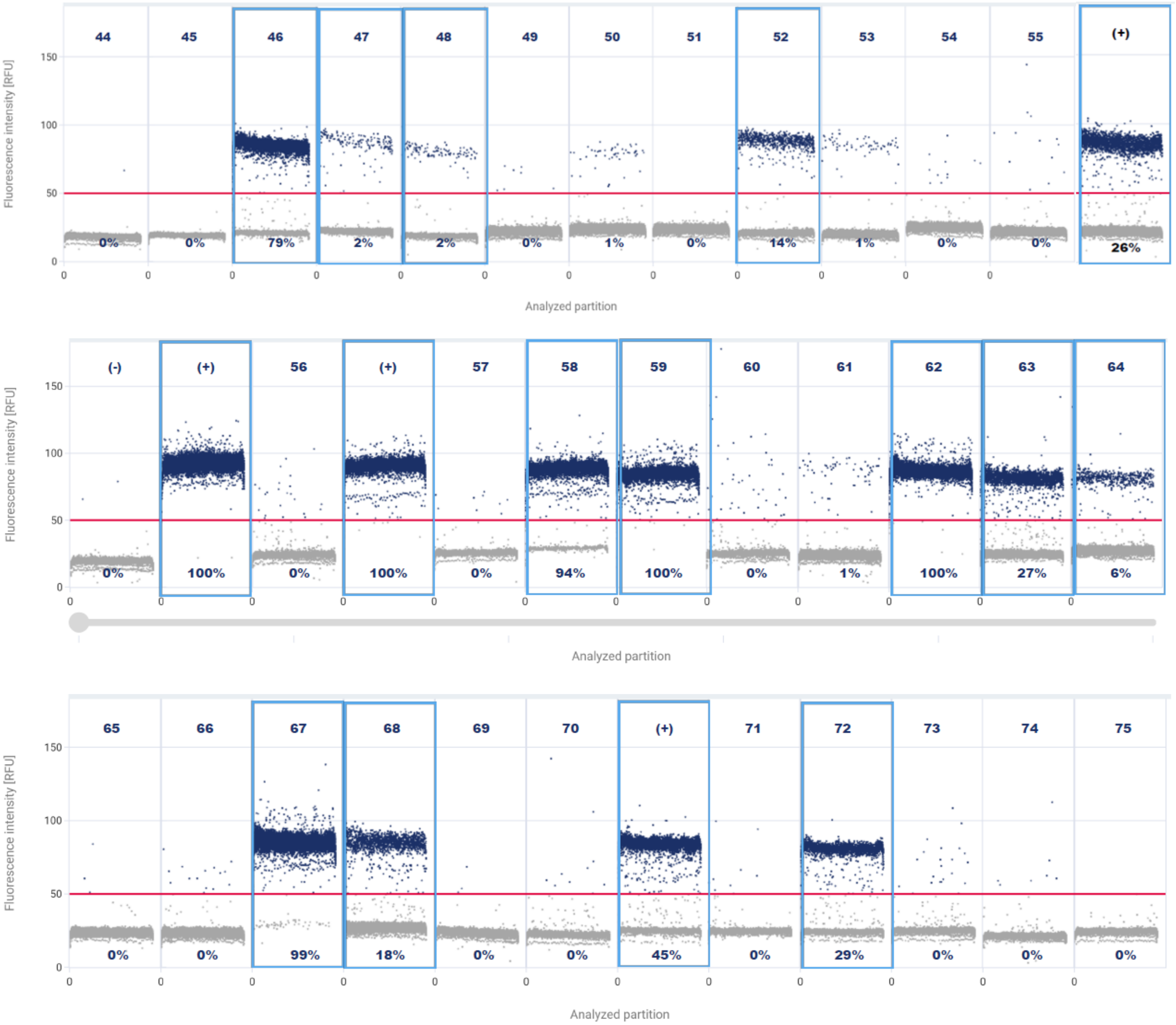
Fluorescence intensity [RFU]
Analyzed partition
44 45 46 47 48 49 50 51 52 53 54 55 (+)
0% 0% 79% 2% 2% 0% 1% 0% 14% 1% 0% 0% 26%
(-) (+) 56 (+) 57 58 59 60 61 62 63 64
0% 100% 0% 100% 0% 94% 100% 0% 1% 100% 27% 6%
65 66 67 68 69 70 (+) 71 72 73 74 75
0% 0% 99% 18% 0% 0% 45% 0% 29% 0% 0% 0%

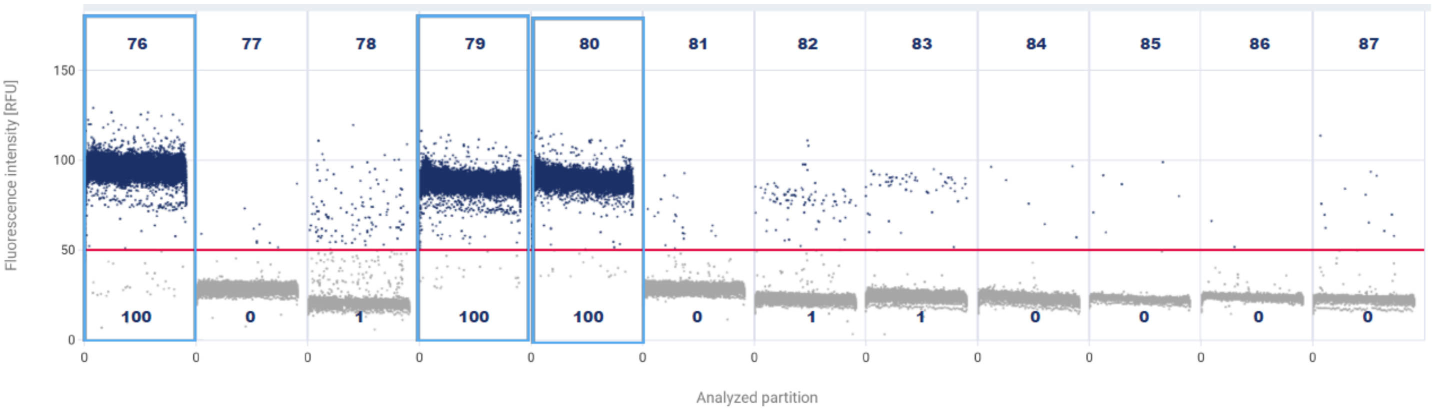
76 77 78 79 80 81 82 83 84 85 86 87
Fluorescence intensity [RFU]
150
100
50
0
100 0 1 100 100 0 1 1 0 0 0 0
Analyzed partition

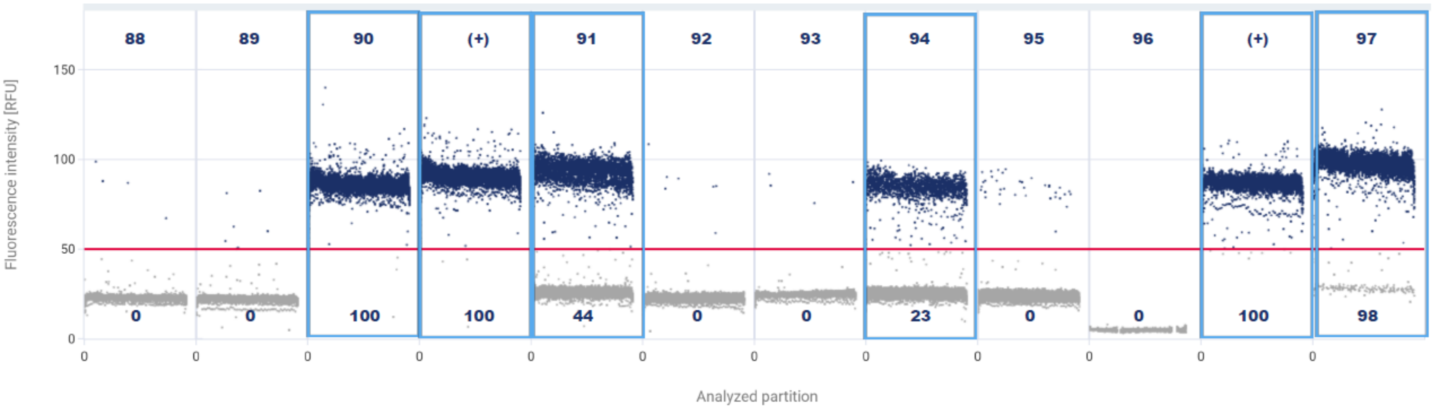
88 89 90 (+) 91 92 93 94 95 96 (+) 97
Fluorescence intensity [RFU]
150
100
50
0
0 0 100 100 44 0 0 23 0 0 100 98
Analyzed partition

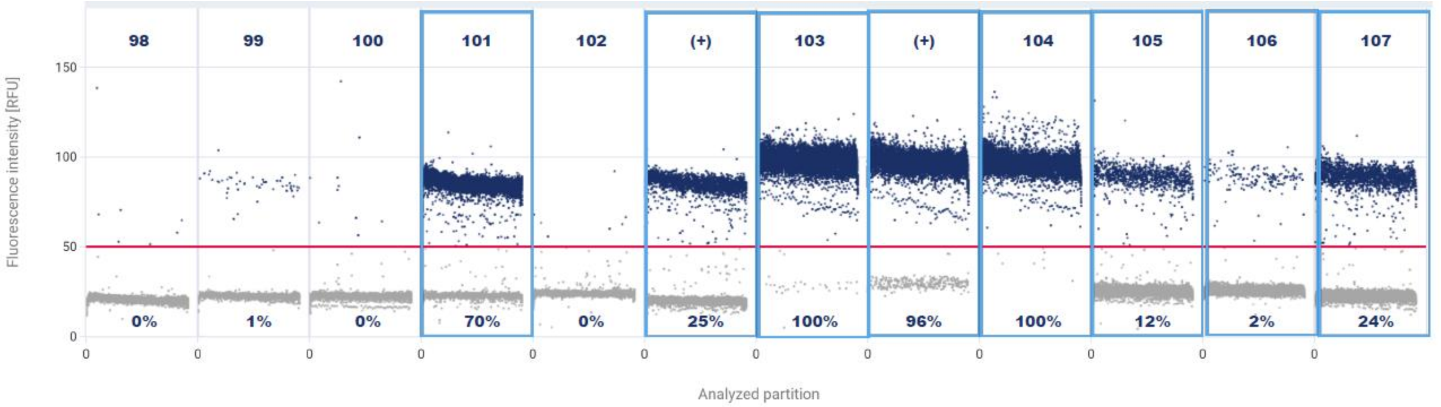
98 99 100 101 102 (+) 103 (+) 104 105 106 107
Fluorescence intensity [RFU]
150
100
50
0
0% 1% 0% 70% 0% 25% 100% 96% 100% 12% 2% 24%
Analyzed partition

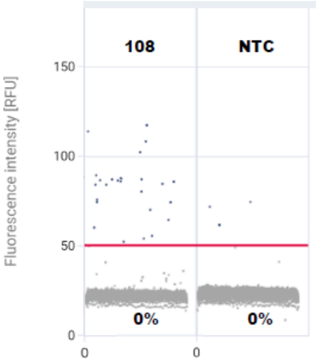
108 NTC
Fluorescence intensity [RFU]
150
100
50
0
0% 0%

TEST 4: Late-Season Leaves (120 DAP)

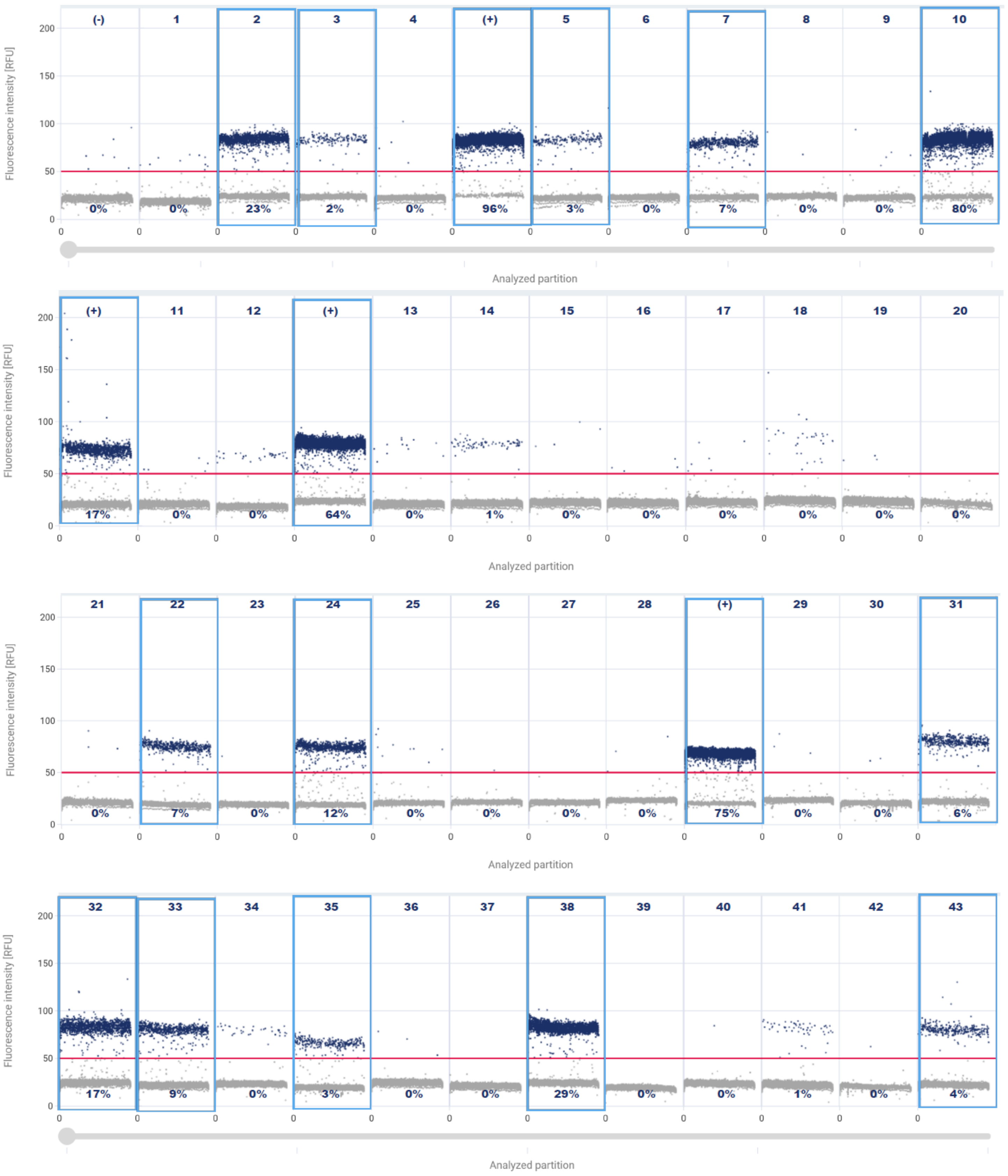

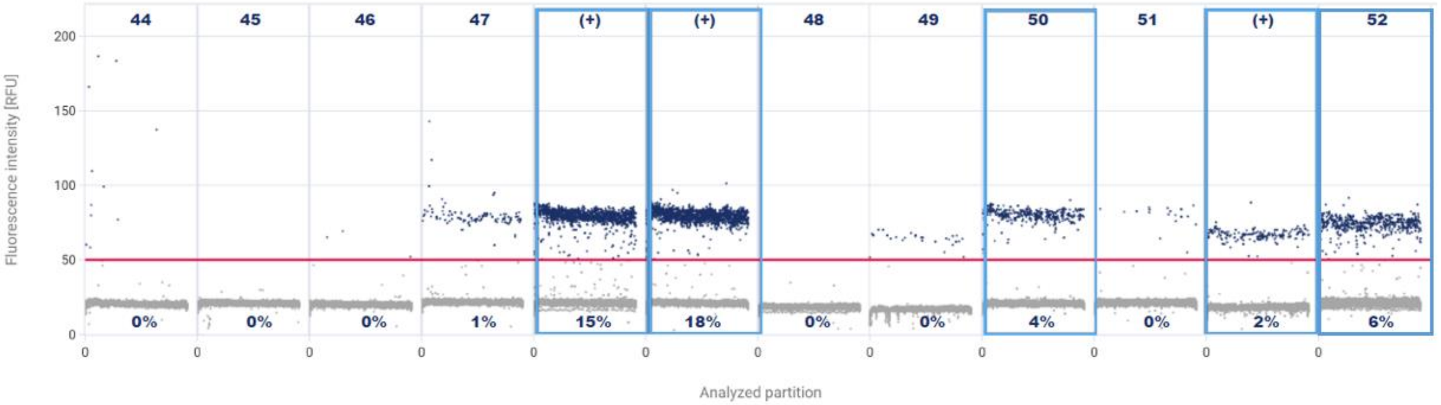
44 45 46 47 (+) (+) 48 49 50 51 (+) 52
Fluorescence intensity [RFU]
200 150 100 50 0
0% 0% 0% 1% 15% 18% 0% 0% 4% 0% 2% 6%
Analyzed partition

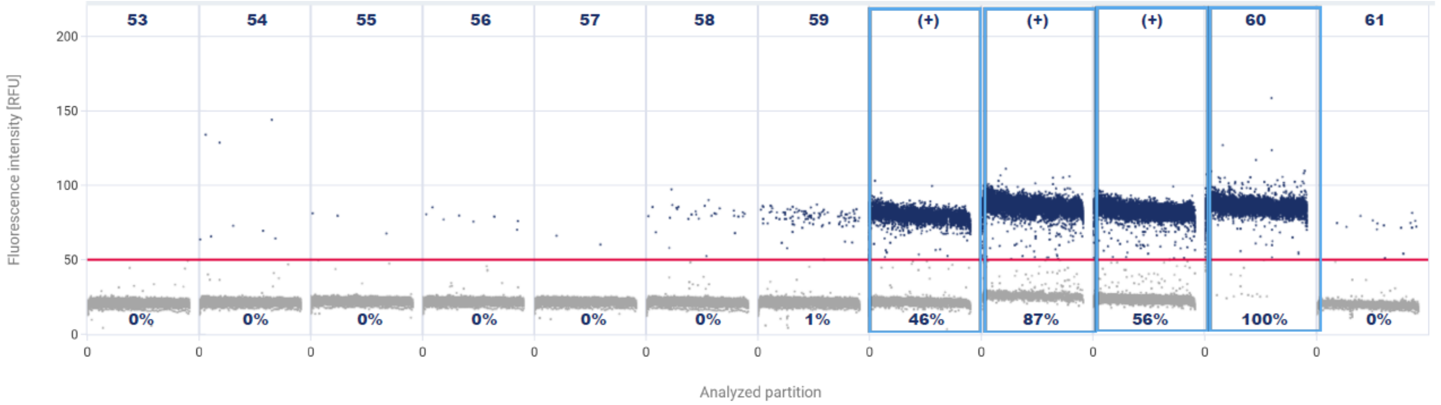
53 54 55 56 57 58 59 (+) (+) (+) 60 61
Fluorescence intensity [RFU]
200 150 100 50 0
0% 0% 0% 0% 0% 0% 1% 46% 87% 56% 100% 0%
Analyzed partition

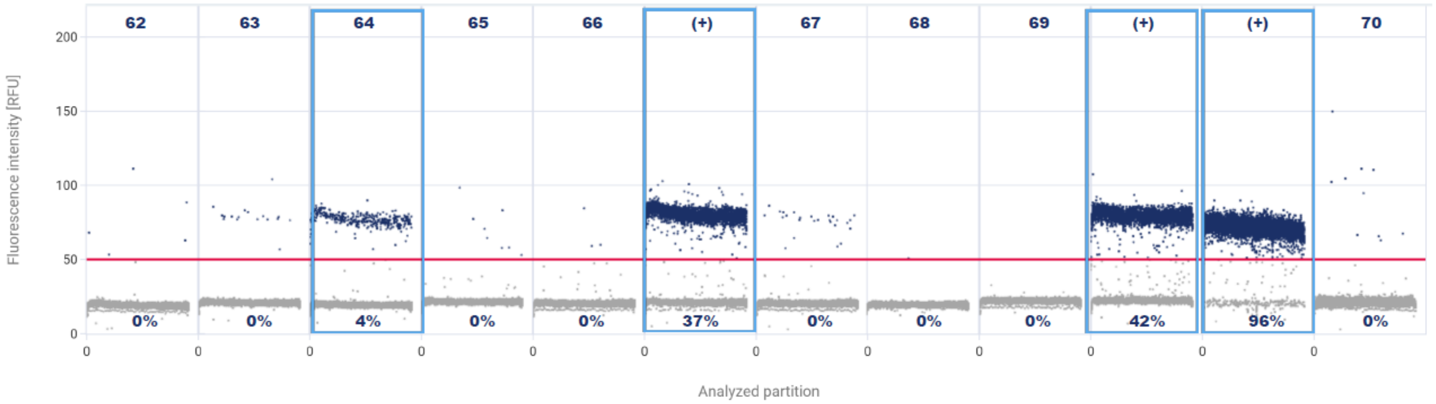
62 63 64 65 66 (+) 67 68 69 (+) (+) 70
Fluorescence intensity [RFU]
200 150 100 50 0
0% 0% 4% 0% 0% 37% 0% 0% 0% 42% 96% 0%
Analyzed partition

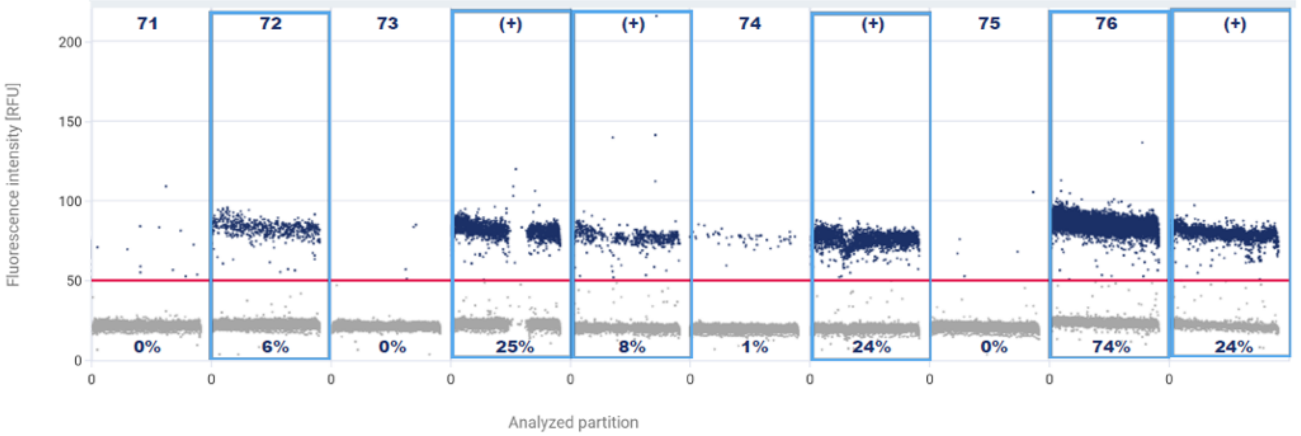
71 72 73 (+) (+) 74 (+) 75 76 (+)
Fluorescence intensity [RFU]
200 150 100 50 0
0% 6% 0% 25% 8% 1% 24% 0% 74% 24%
Analyzed partition

TEST 5: Late-Season Leaves (125 DAP)

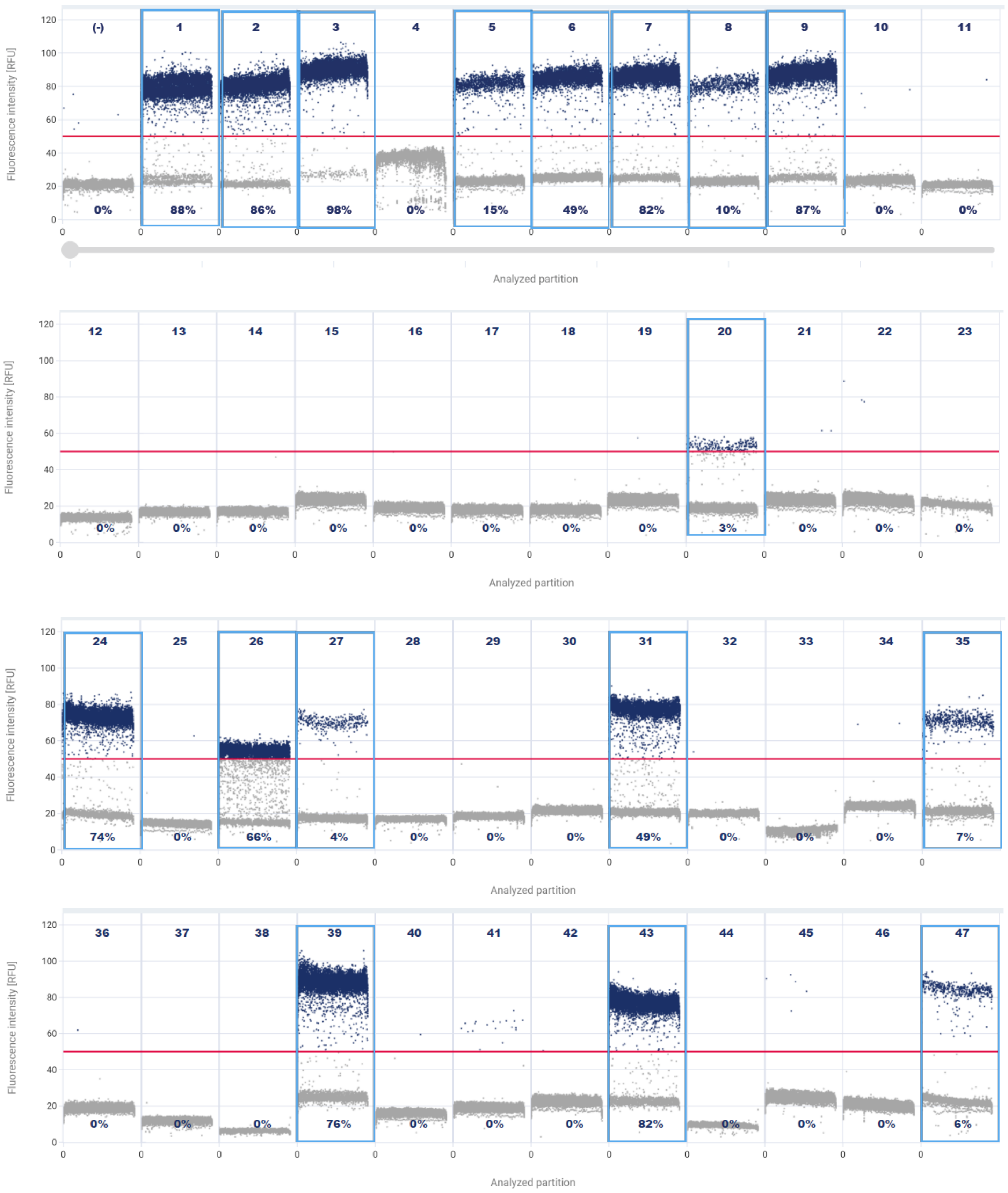

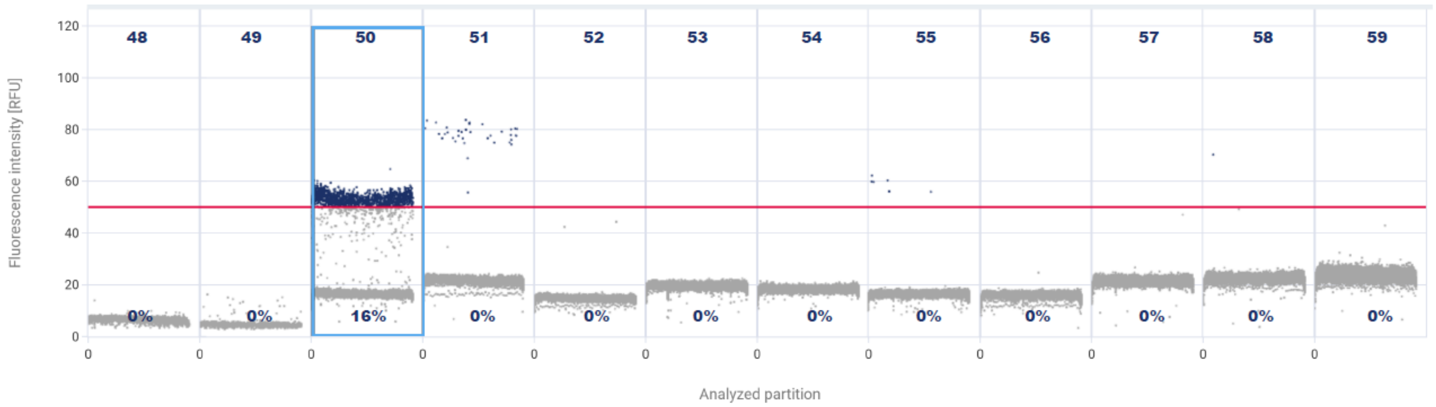
Fluorescence intensity [RFU]
120
100
80
60
40
20
0
48
49
50
51
52
53
54
55
56
57
58
59
0%
0%
16%
0%
0%
0%
0%
0%
0%
0%
0%
0%
Analyzed partition

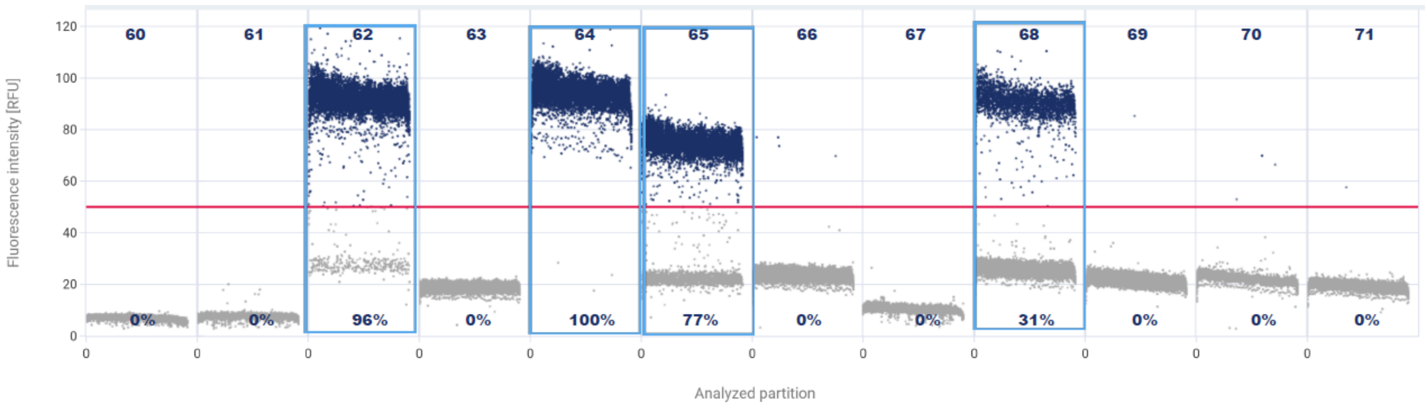
Fluorescence intensity [RFU]
120
100
80
60
40
20
0
60
61
62
63
64
65
66
67
68
69
70
71
0%
0%
96%
0%
100%
77%
0%
0%
31%
0%
0%
0%
Analyzed partition

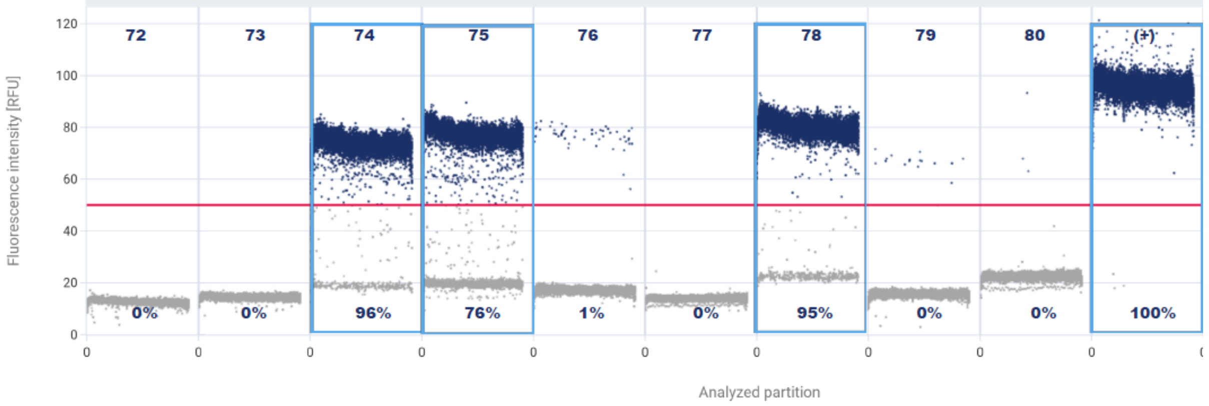
Fluorescence intensity [RFU]
120
100
80
60
40
20
0
72
73
74
75
76
77
78
79
80
(+)
0%
0%
96%
76%
1%
0%
95%
0%
0%
100%
Analyzed partition

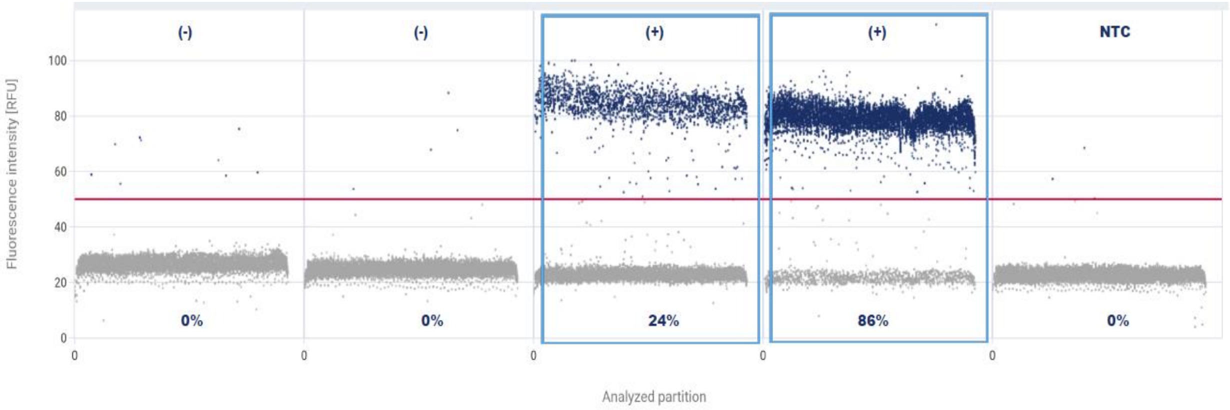
Fluorescence intensity [RFU]
100
80
60
40
20
0
(-)
(-)
(+)
(+)
NTC
0%
0%
24%
86%
0%
Analyzed partition

TEST 6: Late-Season Roots (125 DAP)

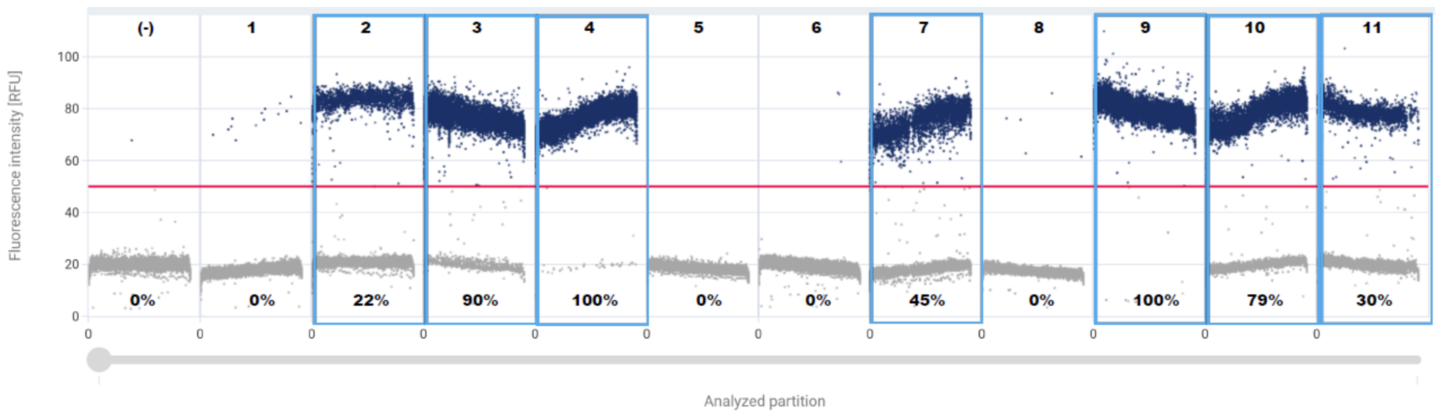

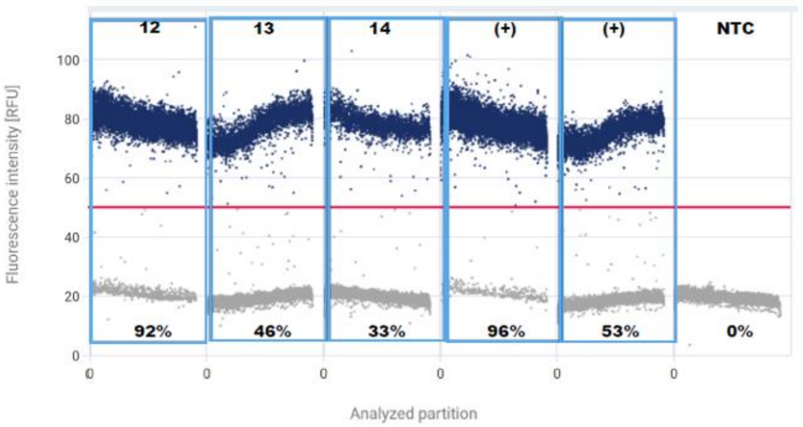

Supplement: Supplementary file 1 [file plants-13-01267-s001.zip › plants-2935482-supplementary.pdf]
